# NTD-DR: Nonnegative tensor decomposition for drug repositioning

**DOI:** 10.1371/journal.pone.0270852

**Published:** 2022-07-21

**Authors:** Ali Akbar Jamali, Yuting Tan, Anthony Kusalik, Fang-Xiang Wu

**Affiliations:** 1 Division of Biomedical Engineering, University of Saskatchewan, Saskatoon, SK, Canada; 2 School of Mathematics and Statistics, Huazhong Normal University, Wuhan, China; 3 Department of Computer Science, University of Saskatchewan, Saskatoon, SK, Canada; 4 Department of Mechanical Engineering, University of Saskatchewan, Saskatoon, SK, Canada; University of Technology Sydney, AUSTRALIA

## Abstract

Computational drug repositioning aims to identify potential applications of existing drugs for the treatment of diseases for which they were not designed. This approach can considerably accelerate the traditional drug discovery process by decreasing the required time and costs of drug development. Tensor decomposition enables us to integrate multiple drug- and disease-related data to boost the performance of prediction. In this study, a nonnegative tensor decomposition for drug repositioning, NTD-DR, is proposed. In order to capture the hidden information in drug-target, drug-disease, and target-disease networks, NTD-DR uses these pairwise associations to construct a three-dimensional tensor representing drug-target-disease triplet associations and integrates them with similarity information of drugs, targets, and disease to make a prediction. We compare NTD-DR with recent state-of-the-art methods in terms of the area under the receiver operating characteristic (ROC) curve (AUC) and the area under the precision and recall curve (AUPR) and find that our method outperforms competing methods. Moreover, case studies with five diseases also confirm the reliability of predictions made by NTD-DR. Our proposed method identifies more known associations among the top 50 predictions than other methods. In addition, novel associations identified by NTD-DR are validated by literature analyses.

## Introduction

Developing a new drug is a costly process in terms of time, risk, and financial resources. For one drug from an initial idea to a product in the market it requires 17–20 years and ~USD 2 billion [1]. Fortunately, complementary approaches can hasten the process of drug discovery. Drug repositioning, wherein an existing approved drug is used to treat a disease other than the one it is designed for, is an opportunity to decrease the relative expense of drug discovery.

Both experimental and computational methods can be used for drug repositioning. Experimental drug repositioning includes screening drugs across a set of targets (i.e. proteins, nucleic acids, etc. which interact with drugs) and diseases that requires facilities and procedures which are expensive and tedious. On the other hand, computational approaches try to avoid these limitations by predicting associations between existing drugs and diseases. The later methods are promising because they are efficient in terms of time, expenses, and results.

Computational drug repositioning methods can be classified into different categories including machine learning, network-based, text mining, and signature matching methods. Most machine learning methods solve the question of drug repositioning as a classification task. Among the many classification algorithms available, support vector machines (SVMs) [2, 3] and random forest [4] have been used for drug repositioning. The most widely used methods for drug repositioning belong to the network-based category, which relies on interaction network data. These methods include random walk [5, 6], matrix factorization [7–11], and network inference [12–15]. Text mining approaches [16, 17] rely on biomedical data and entities, and the co-occurrence of similar/same keywords. Signature matching-based methods for drug repositioning first identify the disease gene signature (drug signature) based on the gene expression profile of the corresponding disease (drug). Then the identified disease gene (drug) signature is connected with drugs (diseases) through the drug-induced gene expression analysis. In this way, the association between drugs and diseases can be identified [18–20].

Regardless of the category, computational methods use experimentally validated associations of drugs and diseases to identify new associations between drugs and diseases. Most of these methods are based on the assumption that if drug C1 is associated with disease D1, and drug C2 is similar to drug C1, disease D1 can be associated with drug C2. Li and Pan [12] used similarity side-information and a hybrid neural network to develop a recommendation system to identify existing and novel associations between drugs and diseases to be used for drug repositioning. Luo *et al*. [21] developed a recommendation system for drug-disease association identification called Drug Repositioning Recommendation System (DDRS). Their method uses drug-disease, disease-condition, and drug-condition networks to determine whether a drug can be used to treat a disease. Xuan *et al*. [8] proposed a nonnegative matrix factorization method called DisDrugPred. They constructed similarity matrices for drugs and diseases based on drug-disease associations. They then used these similarities to predict new associations between drugs and disease. He *et al*. [22] introduced a unique method called PIMD that predicts drug therapeutics using multi-dimensional data. In their method, clusters of drugs that can be used for the treatment of a particular disease are constructed based on multiple drug similarities. Jin *et al*. [13] proposed an approach that integrates multiple heterogeneous networks to predict association scores of drugs and diseases. Their method combines drug and disease features retrieved from multiple drug networks and known drug-disease association networks, respectively.

Despite the advantages of these methods, they usually do not consider drug-disease associations at the molecular level. Any association between drugs and diseases is facilitated by a hidden component or target (gene, protein, etc.) which is typically ignored in drug repositioning methods. To integrate these hidden components in drug repositioning, a method is required that can handle different types of data. A tensor, as an *n*-dimensional array, can store multiple types of association information where each dimension represents one type of data. Tensor decomposition has been used as an effective way to study tensors in various fields of bioinformatics for novel association identification. Huang *et al*. [23] proposed a tensor decomposition method named tensor decomposition with rational constraints (TDRC) to identify the multiple types of associations between microRNAs and diseases. Luo *et al*. [24] developed a tensor decomposition method to identify microRNA-disease associations. In their work, the association information of microRNAs, genes, and diseases was used. To boost the performance of their method, the authors used biological similarity information of microRNAs, genes, and diseases as auxiliary information. Chen *et al*. [25] proposed a tensor decomposition approach to identify drug-target-disease associations for drug discovery. Moreover, Wang *et al*. [26] developed a tensor decomposition method to identify new drug-target-disease triplet associations. Chen *et al*. [27] described a tensor decomposition method named neural tensor network (NeurTN) for personalized medicine. This method combines the concept of tensor and deep neural networks to find the associations among drugs, targets, and diseases. Existing methods suffer from two shortcomings: they consider only triplet associations of drugs, targets, and diseases which ignores valuable pairwise associations; or they use single similarity for drugs, targets, and diseases which ignores the impact of various similarity information. Inspired by this, we use a tensor to integrate triplet association information of drugs, targets, and diseases. In contrast to previous works, we propose a nonnegative tensor decomposition for drug repositioning (NTD-DR) which applies drug-target, drug-disease, and target-disease pairwise associations and combines them to make predictions using multiple types of similarities for drugs, targets, and diseases. Moreover, NTD-DR not only can identify the triplet drug-target-disease associations, but also it can predict the pairwise associations between drugs, targets, and diseases.

**Fig 1:**
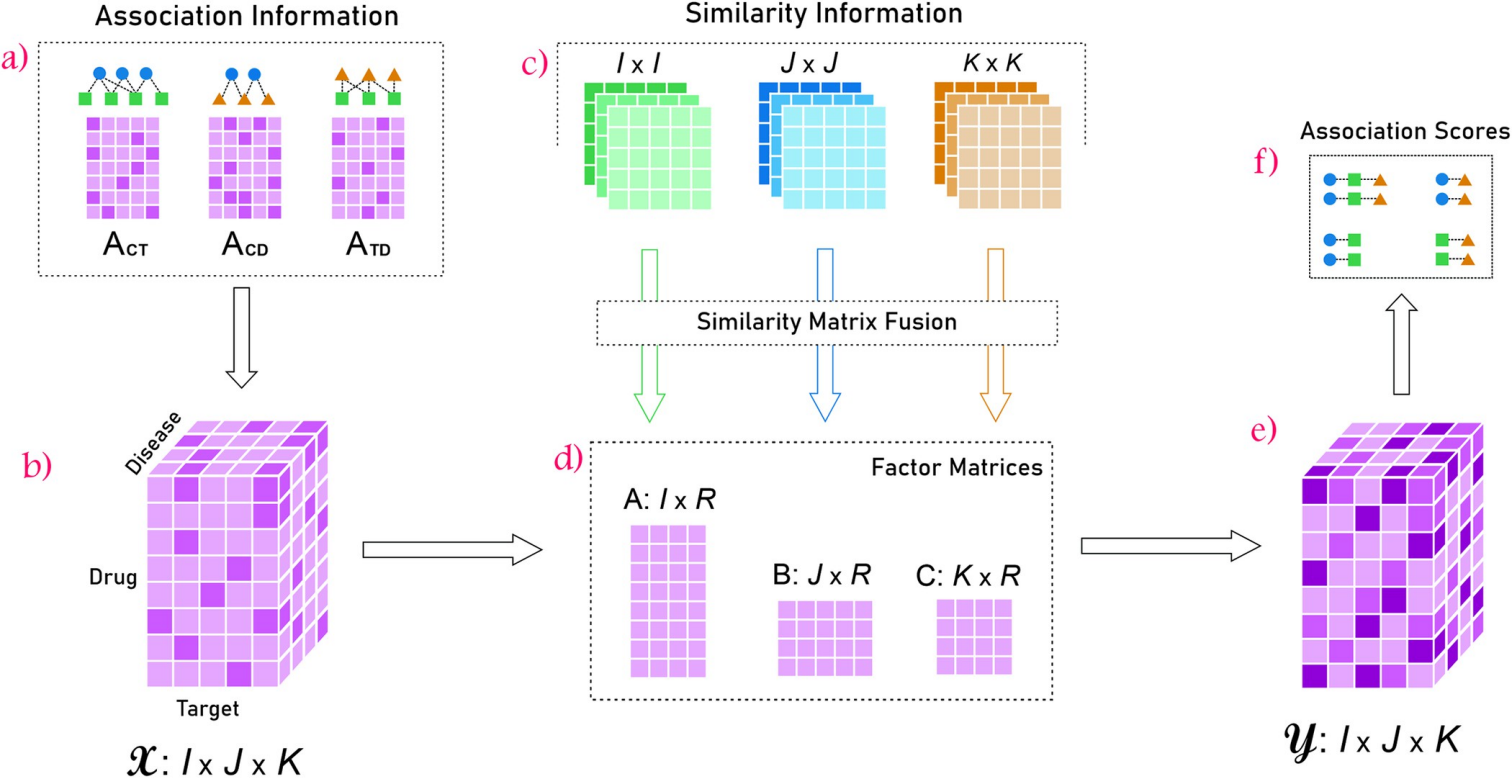
The workflow of NTD-DR. a) Known drug-target (A_CT_), drug-disease (A_CD_), and target-disease (A_TD_) pairwise associations are collected. b) Drug-target-disease association tensor X is constructed based on A_CT_, A_CD_, and A_TD_. c) Multiple similarity measures for drugs, targets, and diseases are collected and are fused to build a single similarity matrix for each of drugs, targets, and diseases. d) Drug-target-disease association tensor is factorized into three factor matrices A, B, and C. e) Tensor Y is reconstructed using similarity matrices upon the convergence of the factor matrices (see Section “Optimization process”). f) The pairwise or triplet association scores are computed.

NTD-DR is outlined as follows. First, we collect drug-disease, drug-target, and target-disease pairwise associations to construct a three-dimensional association tensor. Second, we formulate an objective function to decompose the constructed tensor into three factor matrices and integrate them with similarity information of drugs, targets, and diseases. Then we reconstruct the tensor, based on the factor matrices. Finally, we retrieve the prediction score for triplet and pairwise associations from the reconstructed tensor. We evaluate the performance of our method using cross-validation and separate data. Fig 1 schematically illustrates our algorithm.

## Materials and methods

### Data

#### Association information

We retrieve data from various public sources. To construct a drug-target association matrix, we download data from DrugBank [28], UniProt [29], and SuperTarget [30]. After removing redundant entries, the drug-target association matrix (*A*_*CT*_∈ℝ^*I*×*J*^) consists of 13898 validated associations. Drug-disease associations are downloaded from Online Mendelian Inheritance in Man (OMIM) [31] and the Comparative Toxicogenomics Database (CTD) [32], and *A*_*CD*_∈ℝ^*I*×*K*^ is constructed with 550319 known associations. Moreover, 14730 associations between targets and diseases are retrieved from the Comparative Toxicogenomics Database, OMIM, Uniprot, DisGeNET [33], and GAD [34] to construct *A*_*TD*_∈ℝ^*J*×*K*^.

After the associations between drugs, targets, and diseases are retrieved, we construct the drug-target-disease tensor ($X\in R^{I\times J\times K}$) with 114319 triplet associations. Not surprisingly, our constructed tensor is very sparse (the ratio of known to unknown interactions is 1:119884). The sparsity of the drug-target-disease tensor can lead to the inability of our association models to learn robust feature representations of drugs, targets, and diseases, making them more vulnerable to the cold-start problem, which results in low generalization performance of the models. To mitigate the sparsity of the tensor, in this study we filter out those drugs, targets, and diseases with less than five interactions. After filteration the tensor includes *I* = 810 drugs, *J* = 302 targets and *K* = 542 diseases. This filtration step results in a significant reduction in the sparsity to a ratio of known to unknown interactions of 1:1708. In the final constructed tensor, we randomly divide the validated interactions (known as positive samples) into three subsets: 90% for training and testing our method (dataset P), 5% to set parameters (dataset S), and the remaining 5% as a separate validation data (dataset I) for case studies. All subsets include randomly chosen negative samples equal in number to the positive samples.

#### Similarity information

To boost the performance of prediction we employ different types of similarities for drugs, targets, and diseases to construct multiple similarity matrices for each category. For drugs, we construct five types of similarities, including 1) chemical structure-based and 2) ATC-based similarities using DrugBank data, 3) target-based and 4) gene ontology (GO)-based similarities using UniProt data, and 5) pathway-based similarity using Comparative Toxicogenomics Database data. For targets, we construct three types of similarity matrices: 1) sequence-based similarity, which is computed using sequence structure information of targets retrieved from DrugBank and UniProt; 2) protein-protein interaction (PPI) network similarity of targets, which is calculated based on the data retrieved from InAct [35], BioGrid [36], MINT [37], STRING [38], and HPRD [39]; and 3) gene ontology (GO) semantic similarity of targets, which is computed using data from UniProt. For diseases, we construct four types of similarity matrices, including 1) drug-disease association-based, 2) gene ontology (GO)-based, 3) disease-gene association-based, and 4) PPI-based similarities. These last four similarities are computed using data retrieved from DisGeNET and Comparative Toxicogenomics Database. The construction of these similarity matrices is described in detail in our previous work [40]. Finally, after multiple similarity matrices for drugs, targets, and diseases are constructed, we combine the similarity matrices of each category (drugs, targets, and diseases) via the Similarity Network Fusion (SNF) method described by Wang *et al*. [41] to construct final fused similarity matrices for each category of drugs, targets, and diseases (*S*_*C*_, *S*_*T*_ and *S*_*D*_).

### Problem formulation

This study aims to identify new drug-target-disease, drug-target, drug-disease, and target-disease associations. The identification of these associations can be formulated as a tensor completion problem. Consider a third-order tensor $X\in R^{I\times J\times K}$ to describe known associations among *I* drugs, *J* targets, and *K* diseases, where $X_{ijk}=1$ if the association among drug *i*, target *j*, and disease *k* is known, and 0 otherwise. Assume that the matrix (second-order tensor) *A*_*CT*_∈ℝ^*I*×*J*^ describes known associations among *I* drugs and *J* targets, where *A*_*CTij*_ = 1 if the association between drug *i* and target *j* is known and 0 otherwise. Similarly, the matrix *A*_*CD*_∈ℝ^*I*×*K*^ describes known associations among *I* drugs and *K* diseases, while the matrix *A*_*TD*_∈ℝ^*J*×*K*^ describes known associations among *J* targets and *K* diseases. Furthermore, matrices *S*_*C*_∈ℝ^*I*×*I*^, *S*_*T*_∈ℝ^*J*×*J*^, and *S*_*D*_∈ℝ^*K*×*K*^ describe the similarity of *I* drugs, *J* targets, and *K* diseases, respectively. We use the pairwise associations to construct tensor X and after decomposition, we use similarity information of drugs, targets and diseases to update three factor matrices and reconstruct tensor Y. The rank of tensor Y is the minimum number of rank-1 tensors needed to produce Y as their summation. Therefore, a third-order tensor $Y\in R^{I\times J\times K}$ of rank at most *R* can be written as:

$$Y=\sum_{r=1}^{R}a_{r}\circ b_{r}\circ c_{r}$$ (1)

where *a*_*r*_∈ℝ^*I*^, *b*_*r*_∈ℝ^*J*^, *c*_*r*_∈ℝ^*K*^ for *r* = 1,…,*R*. Elementwise, Eq (1) can be written as:

$$Y_{ijk}=\sum_{r=1}^{R}a_{ir}b_{jr}c_{kr}$$ (2)

for *i* = 1,…, *I*; *j* = 1,…, *J*; and *k* = 1,…, *K*.

The factor matrices refer to the combination of the column vectors from the rank-1 components; i.e., *A* = [*a*_1_,…,*a*_*R*_] and likewise, for *B* and *C*. With this notation, the above third-order tensor can be denoted by Y=[A,B,C].

### Optimization process

Now we consider estimating *A*, *B*, and *C* from data in a third-order tensor X with the constraints that the elements of *A*, *B*, and *C* are nonnegatives. Adopting a least square criterion, we have the following optimization problem:

$$\begin{array}{c} \underset{A,B,C}{\min}\;L_{T}(A,B,C)=\underset{A,B,C}{min}\;\alpha {∥X-Y∥}_{F}^{2}\\ =\underset{A,B,C}{min}\;\alpha ∥X-\sum_{r=1}^{R}a_{r}\circ b_{r}\circ c_{r}{∥}_{F}^{2}\\ s.t.\;A\geq 0,B\geq 0,C\geq 0 \end{array}$$ (3)

where α is a positive regularization coefficient to regulate the tensor decomposition, ${∥Y∥}_{F}^{2}$ is the squared Frobenius norm of the tensor, and the sum of square error is the objective function computed as follows:

$${∥Y∥}_{F}^{2}=\sum_{i=1}^{I}\sum_{j=1}^{J}\sum_{k=1}^{K}Y_{ijk}^{2}$$ (4)

With the concept of the tensor matricization [42], the above objective functions are equivalent to any one of the following functions:

$$\begin{array}{c} L_{T}(A,B,C)=\alpha {∥X_{\left(1\right)}-{A(C⨂B)}^{T}∥}_{F}^{2}\\ L_{T}(A,B,C)=\alpha {∥X_{\left(2\right)}-{B(C⨂A)}^{T}∥}_{F}^{2}\\ L_{T}(A,B,C)=\alpha {∥X_{\left(3\right)}-{C(B⨂A)}^{T}∥}_{F}^{2} \end{array}$$ (5)

where *X*_*(n)*_ is the mode-*n* matricization of tensor X, and ⨂ is the Khatri-Rao product of two matrices. Drug-target, drug-disease, and target-disease pairwise associations are taken into consideration by the following optimization equation:

$$\begin{array}{c} \underset{A,B,C}{\min}\;L_{A}(A,B,C)=\underset{A,B,C}{min}\;\{\lambda_{CT}∥A_{CT}-\sum_{r=1}^{R}a_{r}\circ b_{r}{∥}_{F}^{2}+\\ \lambda_{CD}∥A_{CD}-\sum_{r=1}^{R}a_{r}\circ c_{r}{∥}_{F}^{2}+\lambda_{TD}∥A_{TD}-\sum_{r=1}^{R}b_{r}\circ c_{r}{∥}_{F}^{2}\}\\ =\underset{A,B,C}{\min}\;\{{\lambda_{CT}∥A_{CT}-{AB}^{T}∥}_{F}^{2}+{\lambda_{CD}∥A_{CD}-{AC}^{T}∥}_{F}^{2}\\ +{\lambda_{TD}∥A_{TD}-{BC}^{T}∥}_{F}^{2}\}\\ s.t.\;A\geq 0,B\geq 0,C\geq 0 \end{array}$$ (6)

where *λ*_*CT*_, *λ*_*CD*_, and *λ*_*TD*_ are positive regularization coefficients to regulate the importance of their corresponding associations. The similarity of drugs, targets, and diseases are taken into consideration by the following optimization equation:

$$\begin{array}{c} \underset{A,B,C}{\min}\;L_{S}(A,B,C)=\underset{A,B,C}{min}\;{\{\gamma_{C}\sum_{i,j=1}^{I}S_{Cij}∥a_{i:}-a_{j:}∥}_{2}^{2}+\\ {\gamma_{T}\sum_{i,j=1}^{J}S_{Tij}∥b_{i:}-b_{j:}∥}_{2}^{2}+{\gamma_{D}\sum_{i,j=1}^{K}S_{Dij}∥c_{i:}-c_{j:}∥}_{2}^{2}\}\\ =\underset{A,B,C}{min}\{\gamma_{C}tr(A^{T}L_{C}A)+\gamma_{T}tr(B^{T}L_{T}B)+\gamma_{D}tr(C^{T}L_{D}C)\}\\ s.t.\;A\geq 0,B\geq 0,C\geq 0 \end{array}$$ (7)

where *a*_*i*_: is the *i*-th row of matrix *A*, tr(.) is the trace of a matrix, *L*_*C*_ = *D*_*C*_−*S*_*C*_ is the Laplacian matrix of drugs, *D*_*C*_ is the diagonal matrix whose *i-*th diagonal element is the summation of the *i*-th column of the drug similarity matrix *S*_*C*_, and likewise for *L*_*T*_ and *L*_*D*_. Variables *γ*_*C*_, *γ*_*T*_, and *γ*_*D*_ are positive regularization coefficients to regulate the importance of their corresponding similarities. Then the integrated optimization problem is as follows:

$$\begin{array}{c} \underset{A,B,C}{\min}\;L(A,B,C)=\underset{A,B,C}{min}\;\{L_{T}(A,B,C)+L_{S}(A,B,C)\\ +L_{A}(A,B,C)\}\\ s.t.\;A\geq 0,B\geq 0,C\geq 0 \end{array}$$ (8)

The Karush–Kuhn–Tucker (KKT) conditions [43] for the above optimization problems are similar to those in the work of Tian *et al*. [44] as follows:

$$\begin{array}{c} A\geq 0,\frac{\partial L(A,B,C)}{\partial A}\geq 0,A⋆\frac{\partial L(A,B,C)}{\partial A}=0\\ B\geq 0,\frac{\partial L(A,B,C)}{\partial B}\geq 0,B⋆\frac{\partial L(A,B,C)}{\partial B}=0\\ C\geq 0,\frac{\partial L(A,B,C)}{\partial C}\geq 0,C⋆\frac{\partial L(A,B,C)}{\partial C}=0 \end{array}$$ (9)

Taking the derivatives of L(A,B,C) with respect to *A* yields:

$$\begin{array}{c} \frac{\partial L(A,B,C)}{\partial A}=\frac{\partial L_{T}(A,B,C)}{\partial A}+\frac{\partial L_{S}(A,B,C)}{\partial A}+\frac{\partial L_{A}(A,B,C)}{\partial A}\\ =\alpha (-2X_{\left(1\right)}(C⨂B)+2A{\left(C⨂B\right)}^{T}(C⨂B))+2\gamma_{C}L_{C}A\\ +\lambda_{CT}(-2A_{CT}B+2A(B^{T}B))+\lambda_{CD}(-2A_{CD}C+2A(C^{T}C))\\ =-2(\alpha X_{\left(1\right)}(C⨂B)+\gamma_{C}S_{C}A+\lambda_{CT}A_{CT}B+\lambda_{CD}A_{CD}C)\\ +2A(\alpha {(C^{T}C)⋆(B^{T}B)+\lambda }_{CT}(B^{T}B)+\lambda_{CD}(C^{T}C))+2\gamma_{C}D_{C}A \end{array}$$ (10)

Similarly, the derivatives of L(A,B,C) with respect to *B* and *C* are as follows:

$$\begin{array}{c} \frac{\partial L(A,B,C)}{\partial B}=-2(\alpha X_{\left(2\right)}(C⨂A)+\gamma_{T}S_{T}B+\lambda_{CT}A_{CT}^{T}A+\lambda_{TD}A_{TD}C)\\ +2B(\alpha {(C^{T}C)⋆(A^{T}A)+\lambda }_{CT}(A^{T}A)+\lambda_{TD}(C^{T}C))+2\gamma_{T}D_{T}B\\ \frac{\partial L(A,B,C)}{\partial C}=-2(\alpha X_{\left(3\right)}(B⨂A)+\gamma_{D}S_{D}C+\lambda_{CD}A_{CD}^{T}A+\lambda_{TD}A_{TD}^{T}B)\\ +2C(\alpha (B^{T}B)⋆(A^{T}A){+\lambda }_{CD}(A^{T}A)+\lambda_{TD}(B^{T}B))+2\gamma_{D}D_{D}C \end{array}$$ (11)

Therefore, we can have the following updating rules:

$$\begin{array}{c} A⟵A⋆\left[\frac{{\alpha X}_{\left(1\right)}(C⨂B)+\gamma_{C}S_{C}A+\lambda_{CT}A_{CT}B+\lambda_{CD}A_{CD}C}{A(\alpha (C^{T}C)⋆(B^{T}B)+\lambda_{CT}(B^{T}B)+\lambda_{CD}(C^{T}C))+\gamma_{C}D_{C}A}\right]\\ B⟵B⋆\left[\frac{{\alpha X}_{\left(2\right)}(C⨂A)+\gamma_{T}S_{T}B+\lambda_{CT}A_{CT}^{T}A+\lambda_{TD}A_{TD}C}{B(\alpha (C^{T}C)⋆(A^{T}A)+\lambda_{CT}(A^{T}A)+\lambda_{TD}(C^{T}C))+\gamma_{T}D_{T}B}\right]\\ C⟵C⋆\left[\frac{{\alpha X}_{\left(3\right)}(B⨂A)+\gamma_{D}S_{D}C+\lambda_{CD}A_{CD}^{T}A+\lambda_{TD}A_{TD}^{T}B}{C(\alpha (B^{T}B)⋆(A^{T}A)+\lambda_{CD}(A^{T}A)+\lambda_{TD}(B^{T}B))+\gamma_{D}D_{D}C}\right] \end{array}$$ (12)

where ⋆ is the Hadamard product of two matrices and $\left[\frac{∙}{∙}\right]$ is the element-wise division of two matrices. When matrices *A*, *B*, and *C* converge, tensor association matrices $A_{CT}^{*}$ (drug-target association prediction matrix), $A_{CD}^{*}$ (drug-disease association prediction matrix), and $A_{TD}^{*}$ (targe-disease association prediction matrix) can be constructed based on Eq (2). The aim of this study is to identify the associations between drugs and diseases through the construction of matrix $A_{CD}^{*}$, although our method can also be applied to identify the associations between targets and drugs through $A_{CT}^{*}$ and between targets and diseases through $A_{TD}^{*}$ by appropriately adjusting the parameters in the objective function (8).

### Performance evaluation

In this study, 10-fold cross-validation (CV) is performed in two steps. First, dataset S is used to set up parameters, then dataset P is used to evaluate the performance of the prediction. In the association matrix where the rows (columns) include drugs (diseases), we use experimentally verified associations as positive samples. In most studies, undiscovered associations are used as negative samples. Because these associations can be potentially associations that are not yet discovered, we use the method developed by Luo *et al*. [45] to filter undiscovered associations. Then a number equal to the positive samples in datasets S and P are chosen randomly from filtered undiscovered pairs to be negative samples. Both positive and negative samples are randomly divided into 10 equal subsets. Nine subsets are used in turn as training sets and the remaining subset as test set. CV is performed under three scenarios including:

CV_pairwise_: cross-validation on the pairs within the association matrices, which evaluates the prediction of new pairs by the method.

CV_column-wise_: cross-validation on the columns within the association matrices, which evaluates the prediction of new column (disease) entries. The aim of this CV is to evaluate the performance of our proposed method in detecting associations between existing drugs and diseases that have not been previously treated with those drugs.

CV_row-wise_: cross-validation on the rows within the association matrices, which aims to evaluate the prediction of new row (drug) entries. The aim of this CV is to evaluate the performance of our proposed method in detecting associations between existing diseases and new drugs or drugs that have not been previously used to treat the diseases.

As discussed before, tensor decomposition has the potential to identify novel associations between drug-target-disease, drug-target, drug-disease, and target-disease. As the aim of this study is to focus on the identification of drug and disease associations, we set *λ*_*CD*_ = 1 and *γ*_*C*_ = *γ*_*D*_ = 1. Also, several parameters introduced to our objective functions in Eqs 3–7 are set so as to boost the performance of prediction. Grid search is used to set and select the optimum value for the other parameters. These parameters are as follows: *R*, the rank of the tensor is set from {$\frac{I}{2},\frac{J}{2},\frac{K}{2}$, *I*, *J*, *K*}; *α*, a positive regularization coefficient from {0.1, 0.3, 0.4, 0.7, 0.9}; *λ*_*CT*_ and *λ*_*TD*_ from {10^−1^, 10^−2^, 10^−3^, 10^−4^}; *γ*_*T*_ from {10^−1^, 10^−2^, 10^−3^, 10^−4^, 10^−5^}.

To either set parameters or investigate the performance of the proposed method, the area under the receiver operating characteristic (ROC) curve (AUC) and the area under the precision and recall curve (AUPR) are used as evaluation metrics.

### Implementation

The NTD-DR algorithm is implemented in Python 3.6 programming language using standard modules numpy [46], pandas [47], matplotlib [48], scipy [49], networkx [50], scikit-learn [51]. Comparator methods are also programmed in Python. The source codes and data are available at https://github.com/AliJam82/NTD-DR.

### Case study with a separate dataset

To further investigate the reliability of our method, a separate dataset I as described in Section “Data” is used for case studies. We select a case study disease in dataset I and set its interaction profiles equal to 0. After prediction, we retrieve the association scores of validated associations originally in dataset I. An optimal model should be able to predict greater association scores for validated associations. Since the algorithm is completely blind to this dataset, the accuracy of the predictions made by the method is a reliable measure of the performance of prediction.

## Results

In this section, a comprehensive investigation of the performance of the proposed method is discussed. First, we discuss parameter tuning for our method to obtain an optimal combination of parameters based on the CV on dataset S. Then, we discuss the superiority of the proposed method on recovering missing drug-disease associations by comparing with baseline methods in three CV scenarios using dataset P in terms of AUC and AUPR. Moreover, we present case studies based on the results of an evaluation with a separate dataset, dataset I.

### Parameter optimization

**Fig 2:**
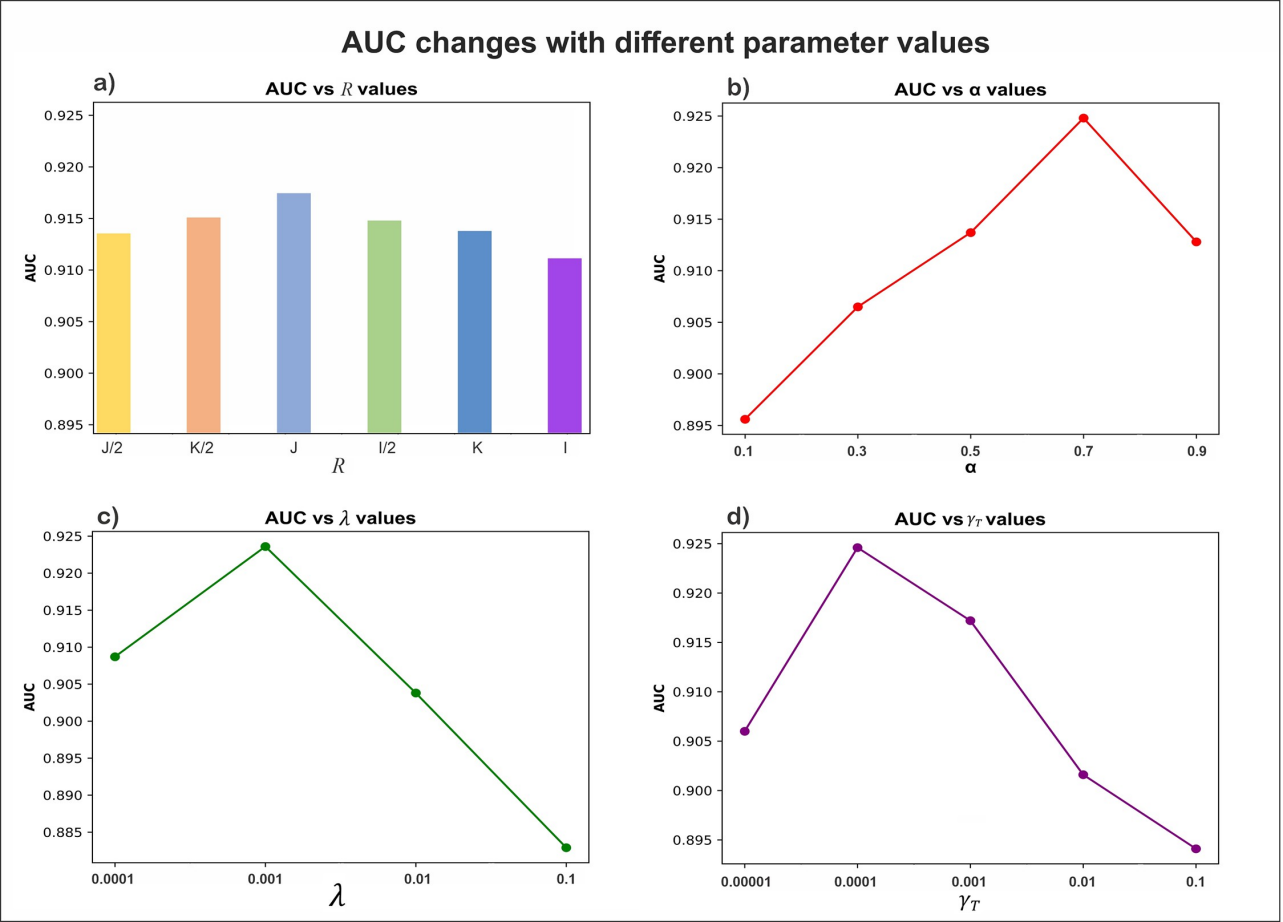
The effect of different values of four parameters on the performance of NTD-DR. The panels show the AUC changes with the increase of parameter a) R b) ∝, c) *λ* (the same value is set to both *λ*_*CT*_ and *λ*_*TD*_), and d) *γ*_*T*_, respectively.

There are several parameters in our proposed method. We analyze each in turn with Scenario CV_pairwise_ yielding the results shown in Fig 2. While Fig 2 only shows AUC, the performance measured AUPR is similar.

#### Impact of *R*

The results (Fig 2A) show that the values for AUC and AUPR generally increase with an increase of *R*. However, the AUC and AUPR start to decrease when *R* exceeds a certain value. The greater values of *R* might lead to an expensive computation and only result in insignificant performance improvement. The best performance is achieved when we set *R* = *J* (the number of targets). Thus, we adopt *R = J* to be the optimal rank of our tensor, which not only increases the performance of prediction but also reduces the cost of computing resources.

#### Impact of *α*

We fix *R* = *J* as the optimal rank of our tensor and vary *α*. As confirmed by the results (Fig 2B), the performance of our method initially improves with increasing *α* and then declines when it is greater than 0.7. The optimal performance achieved when ⍺ = 0.7.

#### Impact of *λ*_*CT*_ and *λ*_*TD*_

We fix ⍺ = 0.7 and *R* = *J* and vary *λ*_*CT*_ and *λ*_*TD*_. We find that (Fig 2C) the performance of our method shows an increasing trend with the decrease of *λ*_*CT*_ and *λ*_*TD*_, but it starts to trend down when *λ*_*CT*_ and *λ*_*TD*_ decrease beyond 10^−3^. The best performance is obtained when *λ*_*CT*_ = *λ*_*TD*_ = 10^−3^.

#### Impact of *γ*_*T*_

We fix other parameters to the above values and vary *γ*_*T*_. The results show (Fig 2D) that both AUC and AUPR values improve with lower values of *γ*_*T*_. However, when the value of *γ*_*T*_ is less than 10^−4^, the performance decreases. The best performance is obtained when *γ*_*T*_ is set to 10^−4^.

### Comparison with other methods

**Fig 3:**
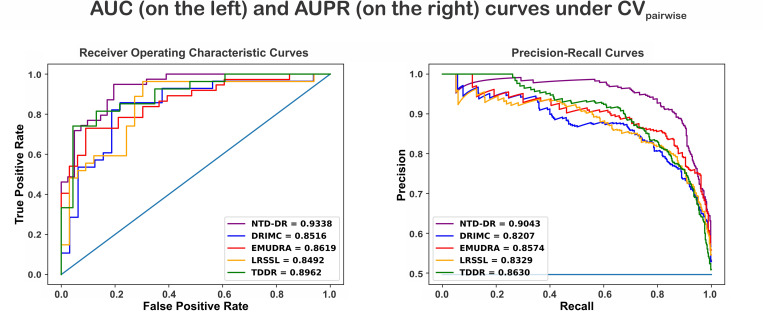
ROC and precision-recall curves of NTD-DR and the existing methods for Scenario CV_pairwise_ using dataset P.

**Fig 4:**
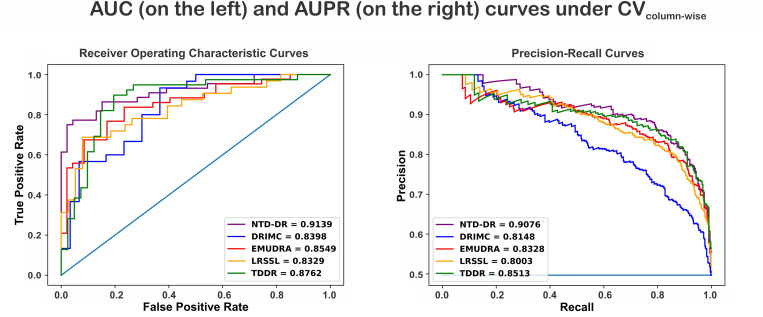
ROC and precision-recall curves of NTD-DR and the existing methods for Scenario CV_column-wise_ using dataset P.

**Fig 5:**
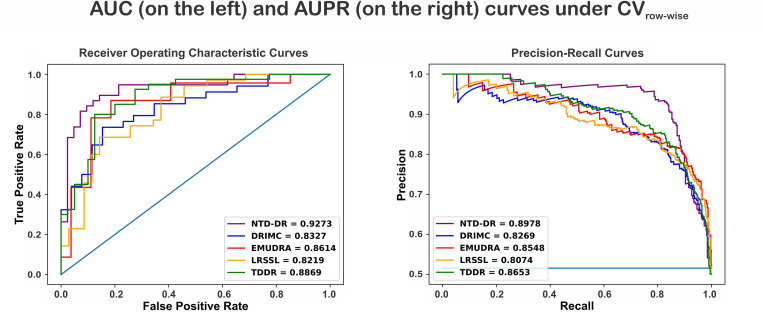
ROC and precision-recall curves of NTD-DR and the existing methods for scenario CVrow-wise using dataset P.

To investigate the performance of our proposed method, we compare its performance in terms of AUC and AUPR with that of existing methods including DRIMC [52], EMUDRA [53], LRSSL [54], and a tensor decomposition method [55] that we refer to as TDDR in this paper. Each method is configured with its defined settings and best parameter values as reported in its original study. Each method is then run with the same data (dataset P) described in Section “Data” using the three cross-validation scenarios. Based on the result and as can be seen in Figs 3–5, our proposed method outperforms all competing methods. Under the CV_pairwise_ scenario, NTD-DR obtains the best performance (AUC = 0.9338, AUPR = 0.9043) compared to that of other competing methods: DRIMC (AUC = 0.8516, AUPR = 0.8207), EMUDRA (AUC = 0.8619, AUPR = 0.8574), LRSSL (AUC = 0.8492, AUPR = 0.8329), and TDDR (AUC = 0.8962, AUPR = 0.8630) (Fig 3). TDDR is another tensor decomposition method and it achieves the second-best results in terms of AUC and AUPR.

The results under the CV_column-wise_ scenario are presented in Fig 4. To summarize, NTD-DR achieves the best performance (AUC = 0.9139, AUPR = 0.9076), outperforming other competing methods: DRIMC (AUC = 0.8398, AUPR = 0.8148), EMUDRA (AUC = 0.8549, AUPR = 0.8328), LRSSL (AUC = 0.8329, AUPR = 0.8003), and TDDR (AUC = 0.8762, AUPR = 0.8513). Under this scenario, TDDR, an alternative tensor decomposition method, shows the second-best performance in terms of AUC and AUPR.

Fig 5 shows the AUC and AUPR values for all competing methods under the the CV_row-wise_. As shown, NTD-DR again obtains the best values for AUC = 0.9273 and AUPR = 0.8978 compared to the other methods: DRIMC (AUC = 0.8327, AUPR = 0.8269), EMUDRA (AUC = 0.8614, AUPR = 0.8548), LRSSL (AUC = 0.8219, AUPR = 0.8074), and TDDR (AUC = 0.8869, AUPR = 0.8653). Similar to the other scenarios, TDDR obtains the second-best results in terms of AUC and AUPR.

### Case study

To further demonstrate the reliability of NTD-DR in discovering novel drug-disease associations, the case studies on breast ductal carcinoma, prostate cancer, pancreatic neoplasms, colorectal neoplasms, and small cell lung carcinoma within dataset I (as described in Section “Case study with an independent dataset”) are performed using the optimal parameter combination determined in Section “Performance evaluation” (i.e., *R* = *J*, ⍺ = 0.7, *λ*_*CT*_ = *λ*_*TD*_ = 10^−3^, and *γ*_*T*_ = 10^−4^). The top 50 predictions according to the predicted association scores, which are drug candidates for the corresponding disease, are shown in S1–S5 Tables. In this study, we assume that if drug C interacts with target T and target T is associated with disease D, then drug C can be associated with the disease D (predicted association) and can be used for the treatment of disease D. We check whether the predictions were in the original dataset I. Those that are not, are deemed novel, hypothesized assocations. We then perform a literature search for evidence to support the novel associations.

For breast ductal carcinoma, NTD-DR predicts 46 experimentally verified associations within its top 50 while the other competing methods predict fewer verified associations. TDDR is the second-best prediction method predicting 37 known associations out of its top 50 predictions. NTD-DR predicts four novel associations with lumiracoxib, etoricoxib, thimerosal, and cisplatin in its top 50 predictions. There is evidence in the literature that the first three drugs can be associated with breast carcinoma via a mutual protein.

#### Lumiracoxib and etoricoxib

The interaction of prostaglandin G/H synthase 2 with lumiracoxib and Etoricoxib were reported by Esser *et al*. [56] and Capone *et al*. [57], respectively. Also, the role of prostaglandin G/H synthase 2 in breast ductal carcinoma was reported by Saindane *et al*. [58]. Therefore, it can be hypothesized that lumiracoxib and etoricoxib are associated with breast ductal carcinoma, as predicted by NTD-DR, through prostaglandin G/H synthase 2.

#### Thimerosal

Stephenson *et al*. [59] found an interaction between thimerosal and superoxide dismutase. On the other hand, Kim *et al*. [60] reported the role of superoxide dismutase in breast ductal carcinoma. We can make a hypothesis that thimerosal is associated with breast ductal carcinoma, as is predicted with NTD-DR, via superoxide dismutase.

#### Cisplatin

There is no evidence in the literature for the association of cisplatin and breast ductal carcinoma. However, it could be that an as yet undiscovered intermediate protein connects cisplatin to this disease and experiments could be performed to find this link.

For prostate cancer, our method predicts 48 experimentally verified associations out of its top 50 predictions. It also identifies two novel associations involving docetaxel and paclitaxel that are supported by the literature.

#### Docetaxel and paclitaxel

Chaudhary *et al*. [61] reported the role of apoptosis regulator Bcl-2 in prostate cancer. On the other hand, interactions of apoptosis regulator Bcl-2 with docetaxel and paclitaxel were discovered by Marshall *et al*. [62] and Gan *et al*. [63], respectively. Therefore, it can be concluded that NTD-DR is justified in predicting associations of prostate cancer with docetaxel and paclitaxel. The second-best method for prostate cancer, TDDR, predicts 41 known associations within its top 50 predictions.

For pancreatic neoplasms, NTD-DR predicts 45 experimentally verified associations and five novel associations with stiripentol, pazopanib, ponatinib, sunitinib, and etoricoxib out of its top 50 predictions. All of these novel associations are supported with literature as follows.

#### Pazopanib, sunitinib, and ponatinib

Dineen *et al*. [64] discovered the association between pancreatic and vascular endothelial growth factor receptor 2. Moreover, Sonpavde *et al*. [65], Mendel *et al*. [66], and O’Hare *et al*. [67] reported interactions between vascular endothelial growth factor receptor 2 and pazopanib, sunitinib, and ponatinib, respectively. NTD-DR is able to identify the association between pancreatic neoplasm and these drugs through the intermediate target.

#### Stiripentol

Fisher *et al*. [68] reported an association between stiripentol and gamma-aminobutyric acid receptor subunit delta. On the other hand, the role of gamma-aminobutyric acid receptor subunit delta in pancreatic neoplasm was reported by Takehara *et al*. [69]. These findings support a hypothesis that stiripentol and pancreatic carcinoma is associated through an intermediate protein, gamma-aminobutyric acid receptor subunit delta.

#### Etoricoxib

The association between etoricoxib and prostaglandin G/H synthase 2 is reported by Capone *et al*. [57]. Moreover, Eibl *et al*. [70] found the role of prostaglandin G/H synthase 2 and pancreatic neoplasm. Based on these findings, we can hypothesize that etoricoxib is associated with pancreatic neoplasm via a mutual target, prostaglandin G/H synthase 2.

TDDR and EMUDRA, as the second-best methods, predict 36 known associations within their top 50 predictions.

For colorectal neoplasms, NTD-DR predicts 50 experimentally verified associations within its top 50 predictions while the second-best method, TDDR predicts 42 known associations within its top 50 predictions. Finally, for small cell lung carcinoma, our method predicts 47 experimentally verified associations and three novel associations with gefitinib, pazopanib, and afatinib within its top 50 predictions that are supported by literature as follows.

#### Gefitinib and afatinib

Sharma *et al*. [71] reported the association between epidermal growth factor receptor and small cell lung carcinoma. On the other hand, the interactions of the epidermal growth factor receptor with gefitinib and afatinib were reported by Ciardiello *et al*. [72] and Masood *et al*. [73], respectively.

#### Pazopanib

The interaction between pazopanib and endothelial growth factor receptor 2 and the association between endothelial growth factor receptor 2 and small cell lung carcinoma were reported by Ciardiello *et al*. [72] and Bonnesen *et al*. [74], respectively.

These findings confirm that our method can predict the associations between small cell lung carcinoma and above-mentioned drugs through corresponding targets. TDDR, as the second-best method, predicts 40 known associations within its top 50 predictions.

The results of these case studies confirm the biological and molecular hypotheses underlying NTD-DR since it can predict the most experimentally verified associations compared to the other methods (Table 1). Moreover, our method can uncover novel associations between drugs and disease implicit in the literature and which are facilitated by a mutual, experimentally verified target.

**Table 1:** The number of known associations in the top 50 predictions made by different methods for different diseases.

	NTD-DR	DRIMC	EMUDRA	LRSSL	TDDR
Breast carcinoma	**46**	32	35	30	37*
Prostate cancer	**48**	36	38	37	41*
Pancreatic neoplasms	**45**	33	36*	31	36*
Colorectal neoplasms	**50**	37	39	36	42*
Small cell lung carcinoma	**47**	34	37	34	40*

The best method in the prediction of the most known associations is in **boldface**, and the second-best method is indicated with *.

## Discussion

In this study, we have proposed NTD-DR, a nonnegative tensor decomposition method, to discover drug-disease associations and enable drug repositioning using triplet associations of drugs, targets, and disease. First, NTD-DR uses pairwise drug-target, drug-disease, and target-disease associations to construct a order-three tensor. Then, to boost the performance of prediction, NTD-DR fuses multiple similarities for drugs, targets, and diseases to construct single similarity measures for drugs, targets, and diseases and later it integrates the similarities of drugs, targets, and diseases with the decomposed tensor to make a prediction. We showed that NTD-DR outperforms existing, alternative state-of-the-art methods. Furthermore, to identify the reliability of NTD-DR, case study analyses were performed. The results confirm that our method can predict a large number of experimentally verified associations in its top 50 predictions. NTD-DR also predicted novel associations. We performed a literature search for evidence to support the novel predictions and found that most are linked together via a mutual target. Although this study focused on the identification of drug and disease associations, the proposed method can investigate associations between drugs and targets, or between targets and diseases by re-setting the value of parameters *γ* and *λ* in the model.

Wet-lab drug-disease and drug-target association identifications are time-consuming. NTD-DR can shorten the duration of these experiments. For instance, NTD-DR can reduce the search space and narrow down the set of drug-target and drug-disease trials to experimentally investigate for drug repositioning. This advantage of NTD-DR makes it a potential filtering approach not only for drug-target interactions, but also for drug-disease associations. We envision various research directions leading from this study. First, to increase the reliability of prediction in terms of biological validation, applying different types of pairwise associations can be used to construct the initial tensor. Second, the tensor decomposition requires huge computational effort for making a prediction, especially when the dimension of the tensor is large. In such a case, a paralleled tensor decomposition would increase the speed of computation. Finally, to make better use of predictions in health care and disease treatment, the predictions need to be validated biologically, experimentally, and pathologically.

In summary, NTD-DR could be effectively used as a reliable method to predict potential associations between drugs and diseases and provide a complementary tool to be used in drug discovery.

## Supporting information

S1 Table The top 50 predictions made by each method for breast ductal carcinoma. (DOCX)

S2 Table The top 50 predictions made by each method for prostate cancer. (DOCX)

S3 Table The top 50 predictions made by each method for pancreatic neoplasms. (DOCX)

S4 Table The top 50 predictions made by each method for colorectal neoplasms. (DOCX)

S5 Table The top 50 predictions made by each method for small cell lung carcinoma. (DOCX)
