# Supplementary material for: NTD-DR: Nonnegative tensor decomposition for drug repositioning
Source: PLoS One. 2022 Jul 21;17(7):e0270852. doi: 10.1371/journal.pone.0270852 (PMC9302855; doi:10.1371/journal.pone.0270852)
Supplement: S1 Table — (DOCX) [file pone.0270852.s001.docx]

S1 Table: The top 50 predictions made by each method for breast ductal carcinoma

|  | NTD-DR | DRIMC | EMUDRA | LRSSL | TDDR |
| --- | --- | --- | --- | --- | --- |
| 1 | **DB00177** | DB00106 | DB00150 | DB00216 | DB00238 |
| 2 | **DB00182** | **DB00136** | **DB00177** | DB00239 | **DB00316** |
| 3 | **DB00188** | DB00155 | **DB00188** | **DB00250** | **DB00317** |
| 4 | **DB00220** | **DB00177** | DB00238 | DB00307 | **DB00361** |
| 5 | **DB00317** | **DB00188** | DB00239 | **DB00316** | **DB00415** |
| 6 | **DB00361** | DB00196 | **DB00250** | **DB00317** | **DB00441** |
| 7 | **DB00398** | DB00197 | **DB00316** | DB00355 | **DB00482** |
| 8 | **DB00412** | **DB00227** | **DB00317** | **DB00361** | DB00498 |
| 9 | **DB00415** | DB00231 | DB00350 | **DB00415** | **DB00523** |
| 10 | **DB00441** | **DB00250** | **DB00361** | **DB00441** | **DB00526** |
| 11 | **DB00471** | DB00343 | **DB00398** | DB00465 | DB00558 |
| 12 | **DB00482** | **DB00398** | **DB00415** | **DB00482** | **DB00559** |
| 13 | DB00515 | **DB00415** | **DB00441** | **DB00559** | **DB00565** |
| 14 | **DB00523** | **DB00441** | DB00459 | DB00560 | **DB00613** |
| 15 | **DB00549** | DB00444 | **DB00482** | **DB00594** | **DB00744** |
| 16 | **DB00594** | **DB00482** | DB00485 | DB00652 | **DB00762** |
| 17 | **DB00744** | DB00490 | **DB00523** | DB00673 | **DB00822** |
| 18 | **DB00762** | **DB00523** | DB00537 | **DB00718** | **DB00843** |
| 19 | **DB00814** | **DB00526** | **DB00549** | DB00728 | **DB00898** |
| 20 | **DB00822** | **DB00559** | DB00569 | **DB00762** | **DB00966** |
| 21 | **DB00843** | **DB00565** | **DB00613** | **DB00822** | **DB01095** |
| 22 | **DB01029** | **DB00594** | DB00658 | DB00826 | **DB01097** |
| 23 | **DB01076** | DB00599 | **DB00718** | DB00908 | **DB01101** |
| 24 | **DB01095** | DB00606 | **DB00762** | DB00931 | **DB01118** |
| 25 | **DB01097** | **DB00744** | **DB00814** | **DB00950** | **DB01128** |
| 26 | **DB01101** | DB00794 | **DB00822** | **DB00966** | **DB01132** |
| 27 | **DB01118** | **DB00814** | **DB00843** | DB01005 | **DB01149** |
| 28 | **DB01128** | DB00931 | **DB00950** | DB01007 | **DB01166** |
| 29 | **DB01136** | DB00949 | **DB00966** | DB01093 | **DB01248** |
| 30 | **DB01236** | **DB01118** | DB01069 | **DB01097** | DB01250 |
| 31 | **DB01238** | **DB01128** | **DB01076** | **DB01101** | **DB01254** |
| 32 | **DB01248** | **DB01136** | **DB01095** | **DB01118** | DB01328 |
| 33 | **DB01254** | **DB01149** | **DB01118** | **DB01128** | DB01501 |
| 34 | **DB01259** | **DB01166** | **DB01128** | DB01154 | DB01606 |
| 35 | **DB01268** | DB01193 | **DB01166** | DB01178 | DB01764 |
| 36 | **DB01283** | **DB01236** | DB01201 | DB01201 | **DB02709** |
| 37 | DB01283 | **DB01254** | **DB01236** | **DB01238** | **DB04855** |
| 38 | **DB01628** | **DB01259** | **DB01254** | **DB01254** | DB04861 |
| 39 | DB01628 | DB01396 | **DB01259** | **DB01259** | **DB05015** |
| 40 | **DB02546** | **DB02546** | DB01327 | DB01319 | **DB06151** |
| 41 | **DB02709** | **DB05015** | **DB02546** | **DB02709** | **DB06176** |
| 42 | **DB04855** | **DB06151** | **DB02709** | **DB04855** | DB06193 |
| 43 | **DB05260** | **DB06176** | **DB05015** | DB05109 | **DB06595** |
| 44 | **DB06176** | **DB06616** | DB05109 | **DB05260** | **DB06603** |
| 45 | **DB06603** | **DB08814** | **DB05260** | **DB06151** | DB06718 |
| 46 | **DB06616** | **DB08881** | **DB06151** | **DB06176** | DB06816 |
| 47 | **DB08814** | DB09238 | **DB06176** | **DB08814** | **DB08814** |
| 48 | **DB08881** | DB09280 | **DB08814** | **DB08881** | **DB08881** |
| 49 | **DB08889** | DB09330 | DB08895 | **DB08889** | DB08897 |
| 50 | DB11590 | **DB11071** | **DB11071** | **DB11071** | **DB11071** |

Experimentally verified targets are indicated in **boldface.**
