# Supplementary material for: NTD-DR: Nonnegative tensor decomposition for drug repositioning
Source: PLoS One. 2022 Jul 21;17(7):e0270852. doi: 10.1371/journal.pone.0270852 (PMC9302855; doi:10.1371/journal.pone.0270852)
Supplement: S2 Table — (DOCX) [file pone.0270852.s002.docx]

S2 Table: The top 50 predictions made by each method for prostate cancer

|  | NTD-DR | DRIMC | EMUDRA | LRSSL | TDDR |
| --- | --- | --- | --- | --- | --- |
| 1 | **DB00091** | **DB00143** | **DB00122** | **DB00114** | **DB00151** |
| 2 | **DB00134** | **DB00169** | **DB00126** | **DB00184** | **DB00162** |
| 3 | **DB00142** | **DB00175** | **DB00142** | **DB00205** | **DB00182** |
| 4 | **DB00175** | **DB00242** | DB00170 | **DB00206** | **DB00205** |
| 5 | **DB00205** | **DB00276** | **DB00184** | **DB00368** | DB00241 |
| 6 | **DB00254** | **DB00277** | **DB00201** | **DB00391** | DB00265 |
| 7 | **DB00255** | DB00278 | DB00239 | **DB00393** | DB00266 |
| 8 | **DB00276** | **DB00297** | **DB00315** | **DB00435** | **DB00295** |
| 9 | **DB00281** | DB00303 | DB00346 | **DB00455** | **DB00321** |
| 10 | **DB00312** | **DB00363** | **DB00363** | **DB00555** | DB00359 |
| 11 | **DB00388** | DB00369 | **DB00398** | **DB00565** | **DB00514** |
| 12 | **DB00393** | DB00419 | DB00419 | **DB00586** | **DB00544** |
| 13 | **DB00421** | **DB00457** | **DB00421** | **DB00586** | DB00592 |
| 14 | **DB00512** | DB00476 | **DB00428** | **DB00625** | **DB00594** |
| 15 | **DB00531** | **DB00502** | **DB00428** | **DB00661** | **DB00633** |
| 16 | **DB00586** | DB00516 | **DB00435** | **DB00759** | **DB00669** |
| 17 | **DB00603** | **DB00541** | **DB00445** | **DB00762** | **DB00669** |
| 18 | **DB00613** | **DB00555** | **DB00477** | **DB00806** | **DB00675** |
| 19 | **DB00657** | DB00572 | **DB00523** | **DB00831** | **DB00695** |
| 20 | **DB00741** | **DB00575** | DB00527 | **DB00863** | **DB00709** |
| 21 | **DB00756** | **DB00590** | **DB00531** | **DB00907** | **DB00740** |
| 22 | **DB00795** | DB00612 | **DB00541** | **DB00914** | **DB00741** |
| 23 | **DB00796** | **DB00625** | **DB00565** | **DB00951** | **DB00773** |
| 24 | **DB00806** | **DB00642** | DB00620 | **DB00958** | DB00788 |
| 25 | **DB00811** | DB00668 | **DB00624** | **DB00997** | DB00872 |
| 26 | **DB00822** | **DB00715** | **DB00642** | **DB00999** | **DB00959** |
| 27 | **DB00834** | DB00727 | **DB00665** | **DB01008** | **DB00975** |
| 28 | **DB00843** | **DB00749** | DB00803 | **DB01029** | DB00989 |
| 29 | **DB00914** | **DB00753** | DB00906 | **DB01076** | **DB01059** |
| 30 | **DB00914** | **DB00759** | DB00980 | **DB01120** | **DB01065** |
| 31 | **DB00997** | **DB00762** | **DB01017** | **DB01128** | DB01079 |
| 32 | **DB01069** | DB00787 | **DB01065** | **DB01149** | **DB01100** |
| 33 | **DB01095** | **DB00966** | **DB01085** | **DB01183** | **DB01119** |
| 34 | **DB01097** | DB00982 | **DB01104** | **DB01259** | **DB01137** |
| 35 | **DB01097** | **DB00987** | **DB01137** | DB01427 | **DB01156** |
| 36 | **DB01104** | **DB01017** | DB01148 | DB01432 | **DB01167** |
| 37 | **DB01110** | DB01018 | **DB01166** | DB02300 | **DB01169** |
| 38 | **DB01120** | **DB01039** | **DB01204** | DB03312 | **DB01216** |
| 39 | **DB01124** | **DB01050** | **DB01234** | **DB06176** | **DB01221** |
| 40 | **DB01197** | DB01061 | **DB01296** | DB06209 | **DB01327** |
| 41 | DB01229 | **DB01095** | DB01298 | DB06698 | **DB01327** |
| 42 | DB01248 | **DB01128** | DB01558 | **DB06774** | **DB01394** |
| 43 | **DB01296** | **DB01136** | **DB02546** | **DB06774** | **DB01396** |
| 44 | **DB01393** | **DB01151** | **DB05239** | DB08815 | **DB06151** |
| 45 | **DB03619** | **DB01174** | **DB06755** | DB08875 | **DB06287** |
| 46 | **DB04224** | **DB01181** | **DB06755** | DB11207 | **DB06603** |
| 47 | **DB05239** | **DB01183** | **DB06777** | DB13595 | **DB06603** |
| 48 | **DB06176** | **DB01254** | **DB08818** | DB13879 | **DB06755** |
| 49 | **DB06616** | **DB03619** | **DB08865** | DB14188 | **DB08901** |
| 50 | **DB08896** | **DB08865** | **DB08896** | DB14725 | **DB13867** |

Experimentally verified targets are indicated in **boldface.**
