# Supplementary material for: NTD-DR: Nonnegative tensor decomposition for drug repositioning
Source: PLoS One. 2022 Jul 21;17(7):e0270852. doi: 10.1371/journal.pone.0270852 (PMC9302855; doi:10.1371/journal.pone.0270852)
Supplement: S3 Table — (DOCX) [file pone.0270852.s003.docx]

S3 Table: The top 50 predictions made by each method for pancreatic neoplasms

|  | NTD-DR | DRIMC | EMUDRA | LRSSL | TDDR |
| --- | --- | --- | --- | --- | --- |
| 1 | **DB00150** | DB00014 | **DB00134** | DB00202 | DB00146 |
| 2 | **DB00175** | DB00245 | **DB00150** | **DB00257** | **DB00206** |
| 3 | **DB00224** | **DB00264** | **DB00188** | DB00340 | **DB00270** |
| 4 | **DB00317** | **DB00276** | **DB00224** | **DB00421** | **DB00307** |
| 5 | **DB00428** | **DB00356** | **DB00268** | DB00442 | **DB00317** |
| 6 | **DB00446** | **DB00367** | **DB00307** | **DB00482** | **DB00333** |
| 7 | **DB00541** | DB00410 | **DB00321** | **DB00526** | **DB00361** |
| 8 | **DB00550** | **DB00421** | **DB00370** | **DB00586** | **DB00376** |
| 9 | **DB00564** | **DB00441** | **DB00398** | **DB00635** | **DB00379** |
| 10 | **DB00573** | **DB00503** | **DB00457** | DB00787 | **DB00441** |
| 11 | **DB00640** | DB00539 | **DB00499** | **DB00841** | **DB00471** |
| 12 | **DB00641** | **DB00559** | **DB00501** | **DB00897** | **DB00501** |
| 13 | **DB00656** | **DB00586** | DB00560 | **DB00912** | **DB00514** |
| 14 | **DB00657** | **DB00637** | DB00615 | **DB00912** | **DB00515** |
| 15 | **DB00679** | **DB00669** | **DB00635** | **DB00993** | **DB00526** |
| 16 | **DB00692** | **DB00715** | DB00651 | DB01012 | **DB00593** |
| 17 | **DB00709** | DB00726 | **DB00668** | **DB01029** | **DB00625** |
| 18 | **DB00741** | **DB00734** | **DB00682** | **DB01032** | **DB00637** |
| 19 | **DB00831** | **DB00738** | DB00701 | **DB01035** | **DB00637** |
| 20 | **DB00834** | **DB00756** | DB00764 | DB01059 | **DB00673** |
| 21 | **DB00841** | DB00906 | DB00839 | **DB01062** | **DB00692** |
| 22 | **DB00857** | **DB01009** | **DB00864** | DB01083 | **DB00795** |
| 23 | **DB00861** | **DB01019** | DB00900 | **DB01115** | **DB00822** |
| 24 | **DB00912** | **DB01039** | DB00978 | DB01116 | **DB00863** |
| 25 | **DB00943** | **DB01039** | **DB01050** | **DB01120** | **DB00864** |
| 26 | **DB01009** | DB01061 | **DB01062** | **DB01132** | DB00949 |
| 27 | **DB01024** | **DB01097** | DB01066 | DB01141 | **DB00975** |
| 28 | **DB01033** | **DB01115** | **DB01088** | **DB01156** | DB00985 |
| 29 | **DB01041** | **DB01165** | **DB01101** | **DB01159** | **DB01016** |
| 30 | **DB01095** | **DB01234** | **DB01120** | **DB01173** | **DB01032** |
| 31 | **DB01119** | DB01243 | DB01147 | **DB01177** | **DB01069** |
| 32 | **DB01128** | **DB01396** | **DB01160** | **DB01199** | **DB01162** |
| 33 | **DB01160** | **DB01407** | **DB01212** | **DB01229** | **DB01165** |
| 34 | **DB01174** | DB01603 | **DB01216** | **DB01248** | **DB01200** |
| 35 | **DB01174** | **DB01213** | **DB01244** | DB01288 | **DB01234** |
| 36 | **DB01213** | **DB03017** | DB01264 | **DB01359** | **DB01241** |
| 37 | DB01268 | DB04815 | **DB01544** | **DB02772** | DB01413 |
| 38 | DB01628 | DB06203 | **DB01563** | **DB04272** | DB01415 |
| 39 | **DB01942** | **DB06204** | DB01623 | **DB06287** | DB01608 |
| 40 | **DB04398** | **DB06755** | DB04822 | DB06690 | DB04812 |
| 41 | DB06589 | DB08864 | **DB04824** | DB08883 | DB06206 |
| 42 | **DB08895** | DB09031 | DB05316 | **DB01213** | DB06212 |
| 43 | DB08901 | **DB09054** | **DB05382** | DB08946 | **DB06403** |
| 44 | **DB08912** | DB09064 | **DB06204** | DB09517 | **DB06616** |
| 45 | **DB08912** | **DB09099** | **DB01213** | DB09571 | DB09049 |
| 46 | **DB09061** | DB09205 | **DB08814** | **DB11672** | **DB09061** |
| 47 | **DB09099** | DB09343 | **DB08916** | DB13284 | DB11323 |
| 48 | DB09118 | DB11064 | **DB09053** | DB13949 | DB12825 |
| 49 | **DB11590** | **DB11590** | **DB09086** | DB14482 | DB13284 |
| 50 | **DB11672** | **DB13867** | **DB11590** | DB14600 | DB14075 |

Experimentally verified targets are indicated in **boldface.**
