# Supplementary material for: NTD-DR: Nonnegative tensor decomposition for drug repositioning
Source: PLoS One. 2022 Jul 21;17(7):e0270852. doi: 10.1371/journal.pone.0270852 (PMC9302855; doi:10.1371/journal.pone.0270852)
Supplement: S4 Table — (DOCX) [file pone.0270852.s004.docx]

S4 Table: The top 50 predictions made by each method for colorectal neoplasms

|  | NTD-DR | DRIMC | EMUDRA | LRSSL | TDDR |
| --- | --- | --- | --- | --- | --- |
| 1 | **DB00142** | DB00157 | **DB00142** | DB00155 | **DB00122** |
| 2 | **DB00205** | **DB00184** | **DB00151** | **DB00169** | **DB00134** |
| 3 | **DB00220** | **DB00213** | **DB00182** | **DB00175** | **DB00193** |
| 4 | **DB00227** | **DB00264** | **DB00238** | **DB00181** | **DB00196** |
| 5 | **DB00247** | **DB00273** | **DB00247** | **DB00188** | **DB00307** |
| 6 | **DB00277** | **DB00297** | **DB00425** | **DB00205** | **DB00334** |
| 7 | **DB00297** | **DB00313** | **DB00435** | DB00217 | **DB00390** |
| 8 | **DB00328** | **DB00363** | **DB00459** | **DB00220** | **DB00412** |
| 9 | **DB00349** | **DB00366** | **DB00582** | DB00240 | **DB00435** |
| 10 | **DB00360** | **DB00477** | **DB00590** | **DB00257** | **DB00440** |
| 11 | **DB00367** | **DB00482** | **DB00625** | DB00258 | **DB00441** |
| 12 | **DB00370** | **DB00490** | **DB00656** | **DB00273** | **DB00446** |
| 13 | **DB00398** | **DB00502** | **DB00673** | **DB00281** | **DB00468** |
| 14 | **DB00445** | DB00504 | **DB00752** | **DB00295** | **DB00550** |
| 15 | **DB00482** | **DB00553** | **DB00794** | **DB00342** | **DB00559** |
| 16 | **DB00532** | **DB00622** | **DB00877** | **DB00358** | **DB00593** |
| 17 | **DB00554** | **DB00640** | **DB00957** | **DB00415** | **DB00677** |
| 18 | **DB00586** | DB00654 | **DB00996** | **DB00444** | **DB00709** |
| 19 | **DB00603** | **DB00678** | **DB01009** | **DB00482** | **DB00800** |
| 20 | **DB00608** | **DB00695** | **DB01035** | **DB00490** | **DB00814** |
| 21 | **DB00622** | **DB00762** | DB01036 | DB00670 | **DB00842** |
| 22 | **DB00636** | **DB00783** | **DB01068** | DB00690 | **DB00857** |
| 23 | **DB00637** | **DB00836** | **DB01097** | **DB00694** | DB00878 |
| 24 | **DB00642** | DB00941 | **DB01118** | **DB00731** | **DB00925** |
| 25 | **DB00682** | DB00981 | **DB01193** | **DB00811** | **DB01009** |
| 26 | **DB00706** | DB01019 | **DB01212** | **DB00822** | **DB01015** |
| 27 | **DB00718** | **DB01043** | **DB01233** | **DB00861** | **DB01015** |
| 28 | **DB00731** | **DB01065** | DB01340 | **DB00908** | **DB01028** |
| 29 | **DB00756** | **DB01119** | **DB01392** | **DB00914** | **DB01035** |
| 30 | **DB00813** | **DB01129** | **DB02530** | **DB00934** | **DB01092** |
| 31 | **DB00842** | **DB01137** | DB04898 | DB00948 | **DB01114** |
| 32 | **DB00914** | **DB01189** | **DB04930** | **DB01113** | DB01145 |
| 33 | **DB00925** | **DB01199** | **DB04951** | **DB01129** | **DB01149** |
| 34 | **DB01011** | **DB01410** | **DB06176** | **DB01162** | DB01340 |
| 35 | **DB01059** | **DB01427** | **DB06403** | **DB01229** | **DB01396** |
| 36 | **DB01092** | DB02638 | **DB06595** | **DB01235** | **DB01400** |
| 37 | **DB01120** | **DB03017** | **DB06616** | **DB01337** | DB01413 |
| 38 | **DB01154** | **DB06800** | DB06706 | **DB01394** | **DB01435** |
| 39 | **DB01186** | **DB08820** | DB06711 | **DB01435** | **DB01435** |
| 40 | **DB01221** | DB08867 | **DB06770** | DB05013 | DB01600 |
| 41 | **DB01238** | DB09072 | **DB06774** | DB05018 | **DB06148** |
| 42 | **DB01242** | DB09143 | **DB08881** | DB06210 | **DB06287** |
| 43 | **DB01244** | **DB09238** | DB08944 | DB06441 | **DB06595** |
| 44 | **DB05381** | **DB09330** | **DB09238** | **DB08907** | **DB08820** |
| 45 | **DB06589** | DB11235 | DB09269 | **DB08910** | **DB08889** |
| 46 | **DB06603** | **DB11750** | DB11340 | **DB09053** | **DB09236** |
| 47 | **DB08910** | **DB11967** | DB12267 | DB09272 | **DB09389** |
| 48 | **DB09070** | DB12127 | DB12792 | DB11431 | DB09534 |
| 49 | **DB09086** | **DB12278** | **DB13179** | DB13211 | DB13163 |
| 50 | **DB12001** | DB12473 | DB13970 | **DB14487** | DB13211 |

Experimentally verified targets are indicated in **boldface.**
