# Supplementary material for: NTD-DR: Nonnegative tensor decomposition for drug repositioning
Source: PLoS One. 2022 Jul 21;17(7):e0270852. doi: 10.1371/journal.pone.0270852 (PMC9302855; doi:10.1371/journal.pone.0270852)
Supplement: S5 Table — (DOCX) [file pone.0270852.s005.docx]

S5 Table: The top 50 predictions made by each method for small cell lung carcinoma

|  | NTD-DR | DRIMC | EMUDRA | LRSSL | TDDR |
| --- | --- | --- | --- | --- | --- |
| 1 | **DB00175** | **DB00091** | DB00115 | **DB00134** | **DB00122** |
| 2 | **DB00193** | **DB00122** | DB00117 | **DB00162** | **DB00175** |
| 3 | **DB00276** | **DB00126** | DB00128 | **DB00193** | **DB00175** |
| 4 | DB00317 | **DB00184** | **DB00152** | DB00218 | DB00192 |
| 5 | **DB00331** | DB00419 | **DB00158** | **DB00328** | DB00250 |
| 6 | **DB00363** | **DB00544** | **DB00175** | **DB00338** | **DB00252** |
| 7 | **DB00381** | **DB00553** | **DB00184** | **DB00342** | **DB00257** |
| 8 | **DB00381** | **DB00563** | **DB00188** | **DB00363** | **DB00412** |
| 9 | **DB00397** | **DB00575** | **DB00252** | **DB00398** | **DB00435** |
| 10 | **DB00415** | **DB00598** | **DB00257** | **DB00457** | **DB00448** |
| 11 | **DB00495** | **DB00633** | **DB00339** | **DB00490** | **DB00490** |
| 12 | **DB00503** | **DB00641** | **DB00342** | DB00529 | **DB00495** |
| 13 | **DB00544** | **DB00642** | **DB00384** | **DB00531** | **DB00499** |
| 14 | **DB00590** | DB00688 | **DB00396** | **DB00559** | **DB00523** |
| 15 | **DB00695** | **DB00694** | **DB00435** | **DB00590** | **DB00526** |
| 16 | **DB00762** | **DB00712** | **DB00445** | **DB00605** | **DB00526** |
| 17 | **DB00762** | **DB00738** | **DB00480** | DB00684 | **DB00531** |
| 18 | **DB00773** | **DB00738** | **DB00526** | **DB00747** | **DB00564** |
| 19 | **DB00841** | **DB00756** | **DB00553** | **DB00758** | **DB00642** |
| 20 | **DB00843** | **DB00796** | **DB00554** | **DB00819** | DB00684 |
| 21 | **DB00907** | DB00813 | **DB00563** | **DB00843** | **DB00773** |
| 22 | **DB00908** | **DB00819** | **DB00571** | **DB00859** | **DB00818** |
| 23 | **DB01017** | **DB00843** | **DB00672** | **DB00877** | **DB00822** |
| 24 | **DB01050** | **DB00877** | **DB00753** | **DB00908** | **DB00860** |
| 25 | **DB01092** | **DB00959** | **DB00758** | **DB00917** | **DB00863** |
| 26 | **DB01095** | **DB00987** | **DB00836** | **DB00917** | **DB00999** |
| 27 | **DB01124** | **DB00993** | **DB00841** | **DB00970** | **DB01050** |
| 28 | **DB01136** | DB01000 | **DB00842** | DB01003 | DB01078 |
| 29 | **DB01174** | **DB01008** | **DB00843** | DB01056 | **DB01118** |
| 30 | **DB01183** | **DB01065** | **DB01136** | **DB01087** | **DB01132** |
| 31 | **DB01235** | **DB01069** | **DB01160** | **DB01110** | DB01147 |
| 32 | **DB01254** | **DB01119** | **DB01169** | **DB01115** | **DB01204** |
| 33 | **DB01268** | **DB01124** | **DB01216** | **DB01118** | **DB01259** |
| 34 | **DB01268** | DB01139 | **DB01393** | DB01162 | **DB01576** |
| 35 | **DB01396** | DB01143 | DB01427 | **DB01234** | **DB01576** |
| 36 | **DB01586** | **DB01149** | DB01624 | **DB01268** | DB01579 |
| 37 | **DB04224** | **DB01956** | **DB01942** | DB01337 | **DB01601** |
| 38 | **DB06176** | **DB02546** | DB04335 | **DB01393** | **DB03793** |
| 39 | DB06589 | DB04209 | **DB05015** | **DB02546** | **DB04930** |
| 40 | **DB06603** | DB06335 | **DB06151** | DB04838 | **DB05015** |
| 41 | **DB06755** | **DB06595** | DB06708 | **DB06151** | **DB06616** |
| 42 | **DB06774** | DB09019 | DB06775 | DB06288 | **DB06755** |
| 43 | **DB06774** | **DB09061** | **DB06777** | DB06702 | DB06782 |
| 44 | **DB06777** | DB09124 | **DB08889** | DB06779 | **DB09061** |
| 45 | **DB08604** | DB09242 | **DB08889** | DB09074 | **DB09070** |
| 46 | **DB08865** | DB09496 | DB09063 | DB09377 | **DB09330** |
| 47 | **DB08901** | DB11061 | DB11133 | DB11800 | **DB11750** |
| 48 | DB08916 | DB11080 | DB11205 | **DB11817** | DB11796 |
| 49 | **DB09086** | DB11636 | DB11589 | DB14206 | DB12554 |
| 50 | **DB11817** | DB13278 | DB14502 | DB14655 | DB15822 |

Experimentally verified targets are indicated in **boldface.**
